# A genome-wide analysis of simple sequence repeats in maize and the development of polymorphism markers from next-generation sequence data

**DOI:** 10.1186/1756-0500-6-403

**Published:** 2013-10-07

**Authors:** Jingtao Qu, Jian Liu

**Affiliations:** 1 Maize Research Institute, Sichuan Agricultural University, Chengdu, Sichuan 611130, China; 2 Key Laboratory of Biology and Genetic Improvement of Maize in Southwest Region, Ministry of Agriculture, Chengdu, China

**Keywords:** Maize, Simple sequence repeat (SSR), Genome, Next-generation sequence, Polymorphism

## Abstract

**Background:**

Maize (*Zea mays ssp*. *mays L*.), as the most important plant for staple food of several million people, animal feed and bioenergy productions, is widely cultivated around the world. Simple sequence repeats (SSRs) are widely used as molecular markers in maize genetics and breeding, but only two thousands pairs of SSRs have been published currently, which hardly satisfies for the increasing needs of geneticists and breeders. Furthermore, the increasing studies have revealed that SSRs also play a vital role in functional regulation and evolution. It is fortunate that the development of sequencing technology and bio-software provides the basis for characterization and development of SSRs in maize.

**Results:**

In this study, MISA was applied to identify overall 179,681 SSRs in maize reference genome B73, with an average distance of 11.46 Kbp. Their distributions within the genome in different regions were non-random, and the density followed in a descending order of UTR, promotor, intron, intergenic and CDS. Meanwhile, 82,694 (46.02%) SSRs with unique flanking sequences were selected, and then applied to analyze the polymorphism of next-generation sequencing data from 345 maize inbred lines and data from maize reference genome B73. There were 58,946 SSRs with length information results in ten or more than ten genomes, accounting for 71.28% of SSRs with unique flanking sequences, while 55,621 SSRs had polymorphism, with an average PIC value of 0.498. 250 pairs of SSR primers in different genomic regions covering all maize chromosomes were randomly chosen for the experimental validation, with an average PIC value of 0.63 in 11 elite maize inbred lines.

**Conclusions:**

Our work provided insight into the non-random distribution spatterns and compositions of SSRs in different regions of maize genome, and also developed more polymorphic SSR markers using next-generation sequencing reads. The genome-wide SSRs polymorphism markers could be useful for genetic analysis and marker-assisted selection in breeding practice, and it was also proved to be high efficient for molecular marker development via next-generation sequencing reads.

## Background

Simple sequence repeats (SSRs) or microsatellites were tandemly arranged repeats of short DNA motifs (1–6 nucleotides long), which extensively distributed in eukaryotes including the plants, animals and microorganisms, as well as in some prokaryotes [1]. SSRs were commonly regarded as genomic “junk” with no significant role as genomic information in a long time until the more utilizing of SSR repeat-number variation and accumulating evidence to support the hypothesis that SSRs could play a positive role in adaptive evolution [2-4]. For assaying genetic variation, SSRs markers based on the repeat number variation were showing significant advantages over the variety of other molecular markers, including restriction fragment length polymorphisms (RFLPs), random amplification of polymorphic DNA (RAPD), and amplified fragment length polymorphisms (AFLPs) [5-7]. As a codominant marker, SSRs have proven to be highly polymorphic, easily reproducible, low costing, facility amplified, and not specifically linked to gene loci of immediate interest [8]. Just for these reasons, SSRs markers turned out to be ideal molecular markers which were widely used in genetic and evolution researches, even as the preferred marker system for many breeding applications. As the development of molecular technology and bioinformatics, increasingly more SSRs with possible functions have been found and characterized, and multiple studies have proved the functional relevance of a significant number of SSRs [2-4,9]. The persistence of intragenic repeats in genomes suggested that there was a compensating benefit [10]. In *Mycoplasma*, a variety of SSRs repeats acted as contingency loci by modulating gene expression or facilitating genome rearrangements via recombination, affecting protein structure and possibly protein-protein interactions, even contributing to the organization of the DNA molecule in cells [9]. Additionally, genes containing intragenic repeats encoded cell-wall proteins in the genome of *Saccharomyces cerevisiae*, which revealed that the variation in intragenic repeat number provided the functional diversity of cell surface antigens allowed rapid adaptation to the environment and elusion of the host immune system in fungi and other pathogens [10]. In humans, allelic differences of SSR repeats numbers were known to cause a wide range of hereditary disorders and disease susceptibilities, such as the ‘triplet repeat diseases’ [2,11]. The presence of SSRs in transcripts of genes in plant species suggested that it might have a role in gene expression and regulation [5,12-14]. The repetitive GCC triplets in the 5’UTRs of ribosomal protein transcripts in maize were believed to influence both gene expression and translation efficiency for the regulation of fertilization [14]. Similarly, SSRs located in the 5’UTR of rice waxy gene were correlated with amylase content [13].

Maize (*Zea mays ssp*. *mays L*.), as the most important plant for staple food of several million people, animal feed and bioenergy productions, is widely cultivated around the world. Therefore, it now poses a serious threat to maize production including yield persistently increasing, quality enhancement, disease and insect damage intensifying and extreme environments. In this situation, it’s urgently needed to strengthen genetic researches and improve breeding efficiency in maize. As a polymerase chain reaction (PCR) is based on efficient molecular markers, SSRs play an important role in maize genetic researches and breeding for a long time. So far, almost two thousands pairs of SSR primers have been published, but they hardly satisfy for the increasing needs of geneticists and breeders. Additionally, the development of SSRs is considerably costly and time consuming through the traditional approaches, but it is fortunate that high-throughput sequencing technologies allow the isolation and development of SSR for more efficient genetic research with high abundance. A series of software for scanning SSRs in the genome have been developed by computational biologists, such as MISA and SSRIT. Benefit from these achievements, the distribution and variation of SSRs frequency were revealed by more researches across species [15-18]. Taking the sequence of maize inbred line B73 as a reference genome, extensive researches are dedicated to structure variation of the genome and transposon identifications [19-21]. With the development of next-generation sequencing technologies, many more cultivars have been analyzed by *de novo* sequencing due to its dramatically low cost and short time. The *de novo* sequencing data from 278 and 86 maize inbred lines were published by Jinsheng Lai and JerMing Chia respectively in August 2012 [22,23], but the distribution and frequency of SSRs in maize genome have not been investigated.

Hence, the goal of this study was to reveal the patterns of SSR distribution in maize genome and explore the database of maize SSR markers to saturate the genetic linkage map. Meanwhile, SSR polymorphism markers were filtered by comparing SSRs in the sequencing data from 345 maize inbred lines and maize reference genome B73. The genome-wide SSRs polymorphism markers could be useful for genetic analysis and marker-assisted selection in breeding practice.

## Results

### Identification and distribution of SSRs in maize genome

A total of 179,681 SSRs were identified on the whole 10 chromosomes, and the average distance between repeat units varied from 11.12 Kb (Chromosome 6) to 11.89 Kb (Chromosome 4), with an average of 11.46 Kb. The detailed information of identified SSRs in maize was summarized in Additional file 1. For the total number of SSRs on each chromosome, Chromosome 1 harbored the maximum number of SSRs (26,718), while Chromosome 10 had the minimum (13,179), which implied that the number of SSRs on chromosome could be positively correlated with chromosome length.

SSRs were distributed in different genomic regions, including promoters, 5’UTR (untranslated region), 3’UTR, CDS (coding sequence), intron, and intergenic regions. As shown in Table 1, most abundance of SSRs was located in the intergenic region (77.25%), while 1.86% was located in the CDS regions. The density of SSRs in different areas of genome varied and followed in a descending order of 5’UTR, 3’UTR, promotor, intron, intergenic and CDS.

**Table 1:** The distribution of SSRs in different areas of genome

**Genome regions**	**Overall SSRs**^**a**^				**Unique SSRs**^**b**^					**Unique SSRs with polymorphisms**^**c**^					**Rate**^**i**^**(%)**
	**Count**	**Interval**^**d**^** (Kbp)**	**Length**^**e**^** (bp)**	**GC**^**f**^** (%)**	**Count**	**Interval**^**d**^** (Kbp)**	**Length (bp)**	**GC (%)**	**Rate**^**g **^**(%)**	**Count**	**Interval**^**d **^**(Kbp)**	**Length (bp)**	**GC (%)**	**Rate**^**h**^** (%)**	
Promotor	14202	5.51	20.98	31.26	11158	7.01	18.98	36.63	78.57	5297	14.78	13.94	24.94	46.42	37.30
5'UTR	3350	4.13	20.93	53.76	3002	4.61	19.64	62.57	89.61	1175	11.79	15.94	45.45	38.59	35.07
3'UTR	3776	5.38	18.18	30.93	3347	6.07	16.61	38.42	88.64	1930	10.52	12.79	21.02	57.13	51.11
CDS	5737	18.79	22.03	70.77	4933	21.85	20.39	80.12	85.99	890	121.12	17.23	64.89	17.86	15.51
INTRON	19232	7.64	19.11	37.89	15367	9.56	17.89	45.34	79.90	7779	18.90	14.18	31.66	49.52	40.45
INTERGENIC	138796	13.67	20.42	43.19	49635	38.23	19.45	41.91	35.76	19955	95.08	13.85	37.33	37.81	14.38
Total^j^	179681	11.46	20.34	42.39	82694	24.90	19.09	43.90	46.02	35046	58.74	13.96	34.38	40.52	19.50

^a^The overall SSRs were identified on the whole 10 chromosomes;

^b^The unique SSRs were SSRs with unique flanking sequences on the genome;

^c^Unique SSRs with polymorphisms were SSRs with length information results in ten or more than ten genomes, and PIC Value ≥ 0.5;

^d^Interval was calculated by number/Kb;

^e^SSR average length was expressed in base pairs (bp);

^f^GC content was evaluated by percentages (%);

^g^This rate was the percentage of unique SSRs against overall SSRs;

^h^This rate was the percentage of unique SSRs with polymorphisms against unique SSRs;

^i^This rate was the percentage of unique SSRs with polymorphisms against overall SSRs;

^j^There were 179681 SSRs in total, due to the alternative splicing occurring in maize genome, and the same SSRs might be divided into different regions and double counting.

Due to over 85% of the maize B73 genome (2.4 Gb) consisted of repetitive DNA, it was necessary to explore the specific SSRs for further researches and applications [19]. There were 82,694 SSRs with unique flanking sequences (unique SSRs), accounting for 46.02% of the entire number of SSRs. Consistent with the total SSRs in genome, the proportion of unique SSRs was located in the intergenic region ranking the highest (56.76%), while 43.24% of the unique SSRs were found in genes (Table 1). Different from the distribution of overall SSRs in genome, although there were an abundance of unique SSRs in intergenic region as well, the density of which was extremely low, with an average distance of 38.23 Kb between two specific loci, far below the density of other regions. In addition, the details of SSRs in different regions were investigated. The result indicated that GC content of SSRs in CDS was up to 80.12%, which was significant higher than any other regions of the genome. Meanwhile, the average sequence length of SSRs in CDS was much longer as well (Table 1).

### Frequencies, repeat sequence length, motif repeats and distribution of different SSR repeat types in maize

The result of detected SSRs by MISA program contained perfect SSRs, compound SSRs and imperfect repeats. Among these three types, perfect SSRs were more abundant with a total number of 166,691 (92.77%). 2,149 (1.20%) SSRs were compound SSRs, containing two or more adjacent motifs in repeats. The imperfect SSRs accounted for 6.03% (10,841), and the repeats of which were interrupted by short tandems. Among the perfect repeats, the most common were MNRs (40.21%), followed by DNRs (29.97%) and TNRs (20.44%). The occurrences of these three SSR types with different repeat unit sizes, a total of 162,826 collectively accounted for 90.62% of the total SSRs. The remaining repeat units, including TTRs, PNRs and HNRs, were made up for 2.15% (3,865) of the total SSRs. The proportion of different SSR types was listed in Table 2.

**Table 2:** The proportion of SSRs with different types

**Types**	**Repeat units**	**Overall SSRs**				**Unique SSRs**				**Unique SSRs with polymorphisms**			
		**Count**	**Length (bp)**	**GC (%)**	**Rate (%)**	**Count**	**Length (bp)**	**GC (%)**	**Rate (%)**	**Count**	**Length (bp)**	**GC (%)**	**Rate (%)**
Perfect SSRs	MNRs	72258	11.70	53.21	40.21	35998	11.79	39.78	43.53	20632	11.20	30.33	58.87
	DNRs	53842	18.24	25.01	29.97	25510	20.38	22.49	30.85	9929	18.54	29.14	28.33
	TNRs	36726	17.52	49.12	20.44	12702	17.83	53.36	15.36	2557	19.04	47.18	7.30
	TTRs	2616	23.08	30.76	1.46	1784	23.45	34.81	2.16	685	24.05	31.43	1.95
	PNRs	800	28.37	41.10	0.45	482	28.05	41.36	0.58	225	27.31	43.09	0.64
	HNRs	449	32.54	51.24	0.25	199	32.38	57.14	0.24	101	31.96	56.41	0.29
	Total^i^	166691	15.41	42.78	92.77	76675	16.08	34.92	92.72	34129	14.35	31.43	97.38
Imperfect SSRs	--	10841	91.66	47.45	6.03	4615	79.28	43.76	5.58	429	40.85	34.12	1.22
Compound SSRs	--	2149	43.48	33.44	1.20	1404	44.53	31.38	1.70	488	34.70	34.34	1.39
Total	--	179681	20.34	42.39	100.00	82694	20.09	36.74	100.00	35046	13.96	34.38	100.0

To assess the contribution of repeat sequence length to SSR abundance, the average length for different types of SSRs was calculated. The total average length of the overall SSRs was 20.34 bp. With regard to different kinds of SSRs, the average length of perfect SSRs, compound SSRs and imperfect SSRs was 15.41 bp, 43.48 bp and 91.66 bp respectively. For perfect SSRs, accounting for the majority of the total SSRs, the length of different repeat unit size varied from 10 bp to 1926 bp and 91.31% of the total SSRs ranged from 10 bp to 50 bp. (AGT)_642_, identified on chromosome 10 (63,833,394-63,835,319 bp), was considered to be the maximum of SSR length (1,926 bp) for perfect SSRs. The average length of HNRs reached 32.54 bp, which was significant longer than the remaining five types of perfect SSRs. Moreover, MNRs had the average length of 11.70 bp, which was the minimum of all SSRs. Furthermore, correlations between the number of observed SSRs and SSR length were taken into account. The results showed that the number of observed SSRs decreased with the increase of SSR length. For the perfect SSRs, the number of observed SSRs also sharply reduced with the increased motif repeats (Additional file 2).

**Figure 1:**
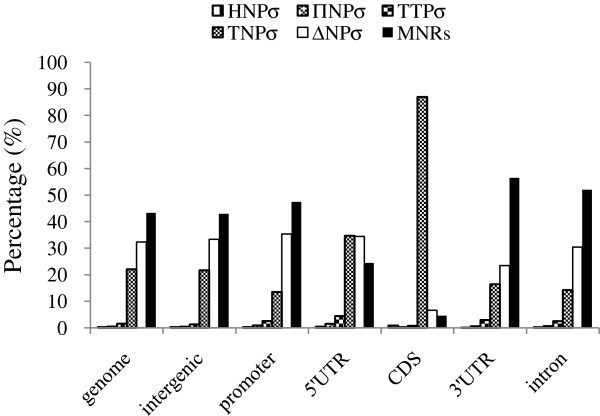
**Percentages of MNRs, DNRs, TNRs TTRs, PNRs and HNRs in different regions of the maize genome.** As shown in the histogram, SSRs detected in all regions decreased except for 5’UTR and CDS with the increase of motif repeats, and TNRs predominated in CDS region.

The distribution of different SSR repeat types was surveyed as well. The major repeat types including MNRs, DNRs and TNRs accounted for almost 90% of the overall SSRs collectively. The distribution proportion of these SSRs in different genomic regions varied (Figure 1). Along with the increase of motif repeats, SSRs detected in all regions decreased except for CDS. In the CDS, MNRs shared only 4.71%, which occupied almost half of the overall SSRs in any other regions. DNRs were analogous to MNRs in CDS with the minimum percentage of 6.72% and evenly distributed in intergenic, while UTRs and intron occupied 34.06%, 30.08% and 31.44% separately. In addition, TNRs predominated in CDS region, accounting for 88.58%, but rarely distributed in any other regions.

### Different repeat units of perfect SSRs in maize

Based on combinations of all four nucleotides, the canonical set of SSR motifs was represented by two different single nucleotides, four different duplets (AC, AG, AT, CG), 10 different triplets, 33 different quadruplets and 102 different quintuplet motifs [24]. All these expected SSR motifs could be represented in maize with variant forms of the same basic set or by their reverse complements. The frequencies of different motifs observed in different areas of the genome were variable. In general, (C/G)n was more abundant (24.44%), followed by (A/T)n (19.94%), (AG)n (16.06%), and (AT)n (12.61%), while the (GC)n motif was the least frequent (1.28%) in maize genome. Of the trinucleotide motifs, (AGC)n was the most abundant (5.14%), followed by (ACG)n (CCG)n (2.81%), (2.61%), (ATC)n (2.49%), (AAG)n (2.48%), (AAT)n (2.07%), (ACC)n (2.04%), (AGG)n (1.22%), (AAC)n (0.85%) and (ACT)n (0.84%) (Table 3). The remaining motifs were present in less than 10% of the total with too many combinations.

**Table 3:** The proportion of SSRs motifs in maize genome

**Motif**	**Total SSRs**			**Unique SSRs**			**Unique SSRs with polymorphisms**		
	**Count**	**Length (bp)**	**Rate (%)**	**Count**	**Length (bp)**	**Rate (%)**	**Count**	**Length (bp)**	**Rate (%)**
A/T	32462	10.96	19.94	22640	10.92	29.18	14221	10.87	42.94
C/G	39796	12.31	24.44	14597	13.14	18.81	6411	11.93	19.36
AT/TA	20534	23.19	12.61	12169	13.26	15.68	3719	19.43	11.23
CG/GC	2085	12.56	1.28	1115	12.77	1.44	157	13.17	0.47
AC/GT/CA/TG	5153	14.05	3.16	3129	14.45	4.03	1318	15.61	3.98
AG/CT/TC/GA	26070	15.63	16.01	10706	17.30	13.80	4735	18.83	14.30
AAC/GTT/TGT/ACA/CAA/TTG	1389	17.93	0.85	1000	18.55	1.29	231	20.40	0.70
AAG/CTT/TTC/GAA/TCT/AGA	4032	16.19	2.48	1263	16.90	1.63	316	18.13	0.95
AAT/ATT/TAA/TTA/TAT/ATA	3378	23.13	2.07	1244	26.59	1.60	297	23.68	0.90
ACC/GGT/CAC/GTG/CCA/TGG	3322	15.85	2.04	1193	16.18	1.54	165	17.58	0.50
ACG/CGT/GAC/GTC/CGA/TCG	4244	17.23	2.61	1255	16.52	1.62	235	17.77	0.71
ACT/AGT/GTA/TAC/TAG/CTA	1372	20.30	0.84	363	17.88	0.47	90	17.83	0.27
AGC/GCT/CTG/CAG/TGC/GCA	8375	16.15	5.14	1773	16.33	2.28	364	17.27	1.10
AGG/CCT/GGA/TCC/CTC/GAG	1981	16.98	1.22	1224	17.47	1.58	327	18.91	0.99
ATC/GAT/CAT/ATG/TCA/TGA	4051	18.15	2.49	777	18.25	1.00	198	20.92	0.60
CCG/CGG/GGC/GCC/GCG/CGC	4582	16.38	2.81	3146	16.66	4.05	334	17.72	1.01
Total	162826	15.15	100	77594	13.94	100	33118	14.01	100

### The number and distribution of unique SSRs with polymorphism

**Figure 2:**
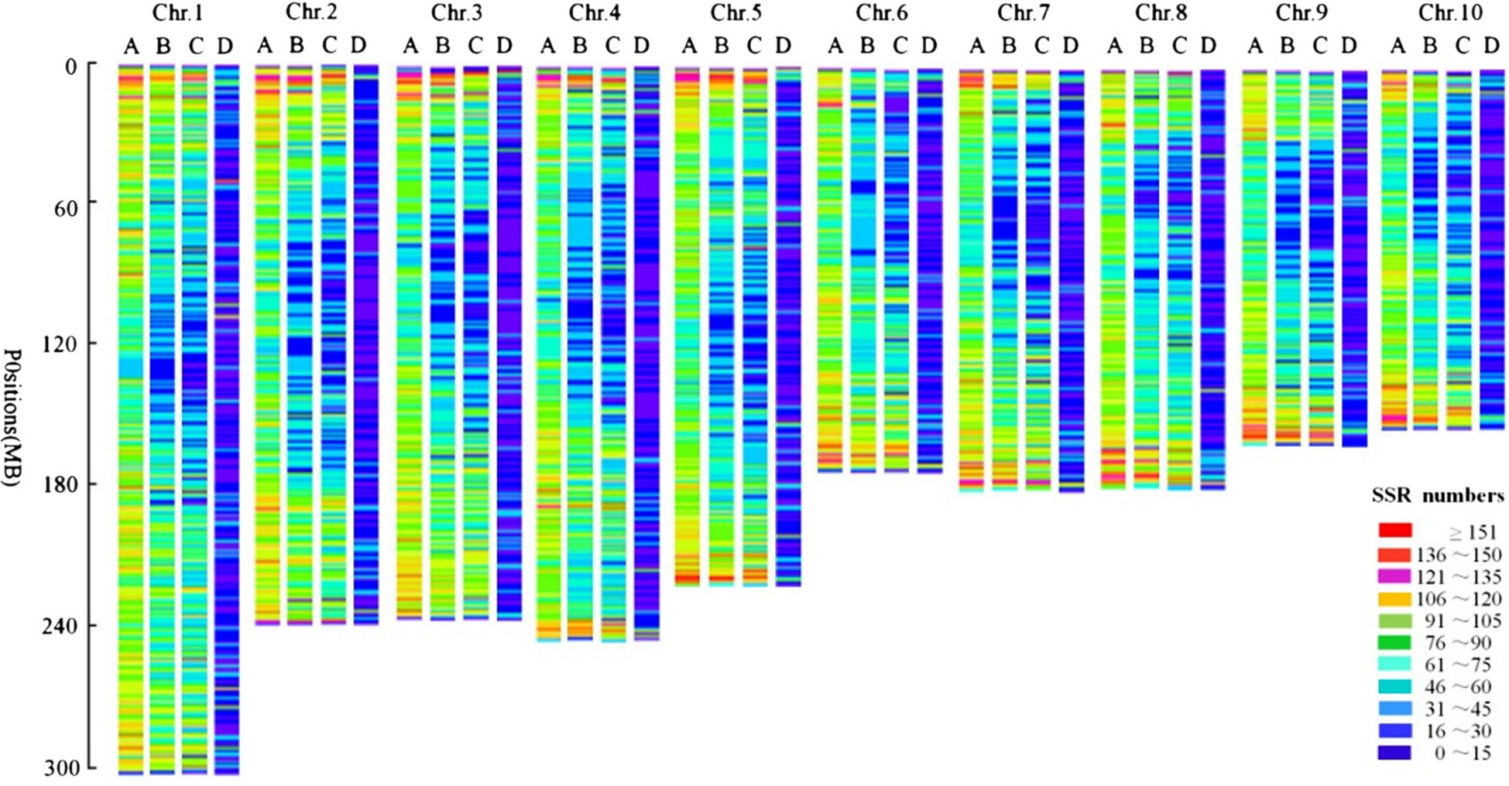
**The distributions for overall SSRs**, **unique SSRs**, **unique SSRs with polymorphism and published SSRs on maize chromosomes. (A)** The distributions for overall SSRs; **(B)** The distributions for unique SSRs; **(C)** The distributions for unique SSRs with polymorphism; **(D)** The distributions for published SSRs (The primers and locus for published SSRs were from MaizeGDB). The identified SSRs were summarized as numbers in 1 Mb bins along each of ten chromosomes.

According to next-generation sequencing data from 345 maize inbred lines, there were 10,527.38 M reads, with average length of 186.02 bp. the sequencing depth of maize inbred line qi410 was the lowest of 0.07×, while the maize inbred line 478 had the highest sequencing depth of 40.25×, with average sequencing depth of 2.89× (Additional file 3). The length information of 346 maize inbred lines, including maize inbred line B73 reference genome, were analyzed by 82,694 SSRs with unique flanking sequences in this study. There were totally 58,946 SSRs with length information results in ten or more than ten genomes, accounting for 71.28% of SSRs with unique flanking sequences, while 55,621 of totally 58,946 SSRs had polymorphism with an average PIC value of 0.498. However, there were 35,046 SSR loci with average PIC value ≥ 0.50, accounting for 42.38% of SSRs with unique flanking sequences (Additional file 4). The distributions for overall SSRs, unique SSRs and unique SSR with polymorphism (PIC ≥ 0.5) on maize chromosomes were shown in Figure 2 (A,B,C). The distribution of SSRs on the chromosomes is non-uniform, and their density in subtelomeric regions tended to be higher than that in the regions nearing to the centromeres, which was in accordance with the distribution of genes in maize [25]. Additionally, the density of unique SSRs with polymorphism was much higher than that of published SSRs in MaizeGDB by comparing unique SSRs (Figure 2B), unique SSRs with polymorphism (Figure 2C) and published SSRs in MaizeGDB (Figure 2D).

In general, with the increase of length differences in SSR sequences, the number of polymorphism SSRs was less. The length discrepancy of SSRs loci ranged from 1 bp to 193 bp. The greatest length discrepancy of 4 bp for SSR locus in different materials predominated with 7,580, accounting for 21.63% of SSR polymorphism locus, while there were 35,928 SSR loci with the greatest length discrepancy ≥ 5 bp, accounting for 64.59% of SSR polymorphism locus. The polymorphism SSRs with more obvious length discrepancy in different genomes were much easier for detecting in experiments (Additional file 4).

For different regions in genome, as shown in Table 1, the unique polymorphism SSRs were most abundant in 3’UTR (57.13%), followed in an order of intronic (49.52%), promotor (46.42%), 5'UTR (38.59%), intergenic regions (37.81%) and CDS (17.86%). It was interesting to note that the polymorphism in genic regions was greater than that in intergenic regions.

### Experimental validation for amplification efficiency and polymorphism of the developed SSR primers

**Figure 3:**
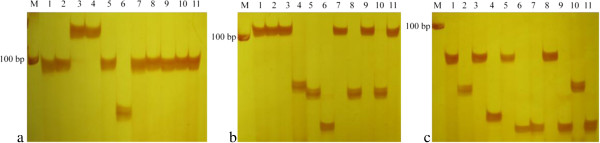
**Experimental validation for three SSRs on chr5**-**206798023 (a), chr4**-**2629366 (b), and chr9-154777915 (c); PCR products from line1 to line11 are B73, Mo17, 91b30, R18, 1212, 583, 3237, R08, Huangzao Si, Zi330, and S37, respectively.** (M: **Marker DL2000**).

250 pairs of primers in different genomic regions covering all maize chromosomes were chosen for the experimental validation. The PIC value of SSR locus in 346 maize genomes ranged from 0 to 0.88, with average PIC value of 0.49. There were 102 SSR polymorphism locus with PIC value < 0.5, with average PIC value of 0.24, while 148 SSR polymorphism locus with PIC value ≥ 0.5, with average PIC value of 0.66. The PIC value of SSR locus in 11 elite maize inbred lines ranged from 0 to 0.89, with average PIC value of 0.63. There were 102 SSR polymorphism locus with PIC value < 0.5, with average PIC value of 0.59, while 148 SSR polymorphism locus with PIC value ≥ 0.5, with average PIC value of 0.654. The alleles detected per primer varied from 2 to 9, with an average of 3.9. (Figure 3).

## Discussion

### The distribution of SSRs in maize

SSRs have been shown to be in both eukaryotic organisms and prokaryotes, with great differences across species in accumulating degree on varied regions of the genome [26]. In eukaryotes, one can expect to encounter at least one simple sequence stretch every 10 kb of DNA sequence [26]. Based on the survey of human genome, one SSR was found to be every 6 kb on average [27]. The SSR frequency in maize was one in every 11.46 Kb, which was lower than that in rice (3.6 Kb) [28]. In general, minor difference was shown for SSR distribution in the same species or similar species. For instance, the distribution of SSRs was very similar with indica and japonica in general, the interval between two SSRs varied from 2.0 kbp to 8.1 kbp, with highly dispersed in 5’ UTR (interval was 2.1 kbp and 2.0 kbp, respectively) and lowly in CDS (interval was 8.1 kbp and 7.7 kbp, respectively) [28]. However, the density of maize SSRs in different genomic regions was unbalanced, ranged from 5.51 kbp (promotor) to 18.79 kbp (CDS). The average GC content in maize SSR sequences (42.39%) was much higher than that in rice genome (27.7%). However, the average length of maize (20.34 bp) was almost equal to that of rice (17.80 bp). For the SSR motif in maize genome, the proportion of MNRs, DNRs, and TNRs was around 40.21%, 29.97%, and 20.44% respectively. However, the great discrepancy in the repeat unions of SSRs revealed that maize was rich in C/G repeats for MNRs, AT repeats for DNRs, and AGC repeats for TNRs, but in rice, A/T, AG and AGG repeats were the most common for the three different types [28].

Intriguingly, the majority of SSRs which resided in CDS were TNRs. Similarly, more than 92% of the predicted SSR within coding sequences had repeat-unit sizes that were a multiple of three in a human cDNA database [29]. The abundance of TNRs in CDS also supported that specific selection against frameshift mutations in coding regions [4,30]. TNRs had not generated frameshifts through expansion of triplet microsatellites, so that which would refrain from selective pressures in coding regions. However, non-triplet microsatellites had to be subject to greater purifying selection with the frameshifts mutations [30]. Therefore, mutation pressure contributed to the enrichment of TNRs in CDS. The strong reading frame and strand preferences were signs of effects of selection, against possible frame shift mutation.

### The polymorphism of SSRs in maize

With the progress of next-generation sequencing technologies, the length of reads in the sequencing results increased gradually from 40 bp initially to 200 bp now. In the *de novo* sequencing data from maize inbred lines, reads with length more than 120 bp accounted for 97.16%. The average length of SSR in maize genome was 20.34 bp, with additional 50 bp in the flanking sequence on both ends, so the average detected length was 70.34 bp. However, the detected SSRs with length lower than 120 bp in SSRs with unique flanking sequences accounted for 97.35%. Thus, most SSR locus could be detected via reads data from *de novo* sequencing, and different sequencing copies at the same loci could be used as repeats for enhancing data accuracy. Maize inbred line Mo17 was partly sequenced by Roche 454 technology, with average length of 400 bp, and 39,274 (47.49%) SSR locus were detected in Mo17 genome. The detection rate of SSR locus in maize inbred lines with similar sequencing depth, such as zheng58, reached 46%, which indicated that the detection rate depended on the sequencing depth. According to the length, 94.37% of SSR locus could be detected in the genome, but only 47.49% of SSR locus was detected in the genome of Mo17, a maize inbred line with the most results. SSR locus without detection results were mainly caused by the base discrepancy of flanking sequences.

SSRs were widely concerned and used as an ideal tool for deciphering genetic variability, not only due to the abundance within a genome, the random occurrence, but also the high degree of polymorphisms [27]. The analysis of SSR polymorphism locus in the sequencing data from 346 maize inbred lines revealed that SSR locus in maize genome had extensive polymorphism. According to the published researches on genetic diversity in maize inbred lines, the average PIC for SSR markers in different studies varied from 0.47 to 0.69, with a mean value of 0.607, which was in agreement with the results [31-41]. The results from experimental validation were slighter higher than that from 346 genomes. Unique SSR locus were selected for the alignment of SSR polymorphism locus via software, and each loci was only one value in each genome. However, there are always several bands in one material due to non-specific amplification during the experiment, which also increases SSR polymorphism locus in the detection. The developed SSR locus with great length discrepancy, high polymorphism and density might have a higher chance of polymorphism exhibition in populations. Therefore, SSRs primers are especially important and efficient for practical application value in genetic researches and molecular breeding.

Two points were noteworthy for unique SSRs with polymorphisms in maize. Firstly, the variation level in maize inbred lines was relatively high, and the polymorphism rate of model maize inbred lines between B73 and Mo17, accounting for 66.04%, was even higher than that of rice subspecies, accounting for 51.80% [28]. Secondly, the polymorphism in intragenic regions of maize genome was higher than that in intergenic regions, while the opposite result was showed in rice.

Maize is a kind of species with high domestication and artificial selection, so it completely depends on humans for its survival, which leads to the fact that researchers cannot find the progenitor for maize in a long time. Hybrid maize breeding took full advantage of heterosis, and it was produced by inbred lines that originated from divergent heterotic groups. The greater the genetic variation in maize inbred lines was, the more obvious the heterosis phenomenon was. In the view of SSR polymorphism, the genetic variation in maize inbred lines was close to different rice subspecies, which also reflected that maize was a highly polymorphic species [42]. The results showed that the polymorphism of SSR was affected by artificial selection in the process of maize breeding. This selection aimed to function also directly showed that SSR polymorphism with 44.31% in intragenic regions of maize was higher than that with 37.81% in intergenic regions. Furthermore, the diversity of 462 SSRs in maize genome and its wild progenitor, teosinte were observed to reveal how the domestication bottlenecks and artificial selection shaped the amount and distribution of genetic variation in maize genome [43].

## Conclusions

Our work provided insight into the non-random distribution spatterns and compositions of SSRs in different regions of maize genome, and also developed more polymorphic SSR markers using next-generation sequencing reads. The genome-wide SSRs polymorphism markers could be useful for genetic analysis and marker-assisted selection in breeding practice, and it was also proved to be high efficient for molecular marker development via next-generation sequencing reads.

## Methods

### Maize genome sequence sources

The genome sequences for maize B73 (Release ZmB73_RefGen_v2) and Mo17 (454 pyrosequencing data) were downloaded from http://www.maizesequence.org/index.html and http://www.phytozome.net/maize.phprespectively. 5’UTR, coding determining sequences (CDS), 3’UTR, exon, intron and intergenic regions were provided by the annotation of B73 genome (ZmB73_5b_FGS, http://ftp.maizesequence.org/current/filtered-set/). The genomic DNA sequences of 2 Kb from upstream of star codon (ATG) were analyzed as promoters. The de novo sequencing data of 345 maize materials were downloaded from NCBI (http://www.ncbi.nlm.nih.gov/sra?term=SRA049859 and http://www.ncbi.nlm.nih.gov/sra?term=SRA051245), including 151 elite Chinese lines, 88 Ex-PVP lines, 50 improved maize lines, 23 maize landraces, 33 public US lines (Additional file 3).

### SSRs screening in maize reference genome B73

Microsatellite search module (MISA), a SSRs motif scanning tool written in Perl (http://pgrc.ipk-gatersleben.de/misa/), was used for the identification and localization of perfect microsatellites, compound microsatellites and imperfect microsatellites which were interrupted by a certain number of bases [44]. The identified motifs were one to sixnucleotides in size, and the minimum repeat unit was defined as 10 for mononucleotides (MNRs), seven for di-nucleotides (DNRs), six for tri-nucleotides (TNRs), five for tetra-nucleotides (TTRs) and four for all the higher order motifs including penta-nucleotides (PNRs) and hexa-nucleotides (HNRs). Furthermore, the maximal number of interrupting base pairs in a compound microsatellite was 100 bp. The variation and reverse complement of each motif were categorized into the same groups.

### Identification of SSRs with unique flanking sequences

The first 20 bp sequences of 5 bp in upstream of SSR loci were extracted by program written in Perl as the upstream primer of e-PCR, while the last 20 bp sequences of 5 bp in downstream of SSR loci were extracted as the downstream prime after inverted repeats. These primer sequences with Bowtie software were aligned against to maize reference genome, allowing up to one mismatched, and then SSRs with unique flanking sequences were identified by program written in Perl [45].

### Variation of SSRs in 346 maize inbred lines

Taking the maize reference genome sequence and reads of the *de novo* sequencing data from 345 maize inbred lines as the template, the sequences around SSRs with unique flanking sequences were aligned against via Bowtie to extract the length information about SSR loci by program written in Perl. The allelic diversity of each SSR locus was assessed by the polymorphism information content (PIC), which is defined as PIC_*i*_ = 1- $\overset{n}{\underset{j=1}{\sum }}p_{\mathrm{ij}}^{2}$, where p_*ij*_ is the frequency of the *j*th pattern for the *i*th markers. Furthermore, the length of polymorphism SSRs was investigated in the reference genome [46].

### PCR-based primer design

The unique hits were selected for primer design. The sequences include SSR motif and two 100 bp flanking sequences on each side of the repeat were used for automatically primer designed by Primer3 [47] through following parameters: primer length range from 20 nt to 28 nt, with optimum 23 nt; melting temperature (Tm) of 60°C to 65°C, with optimum temperature of 63°C, and primer pairs must have a similar Tm value with GC content around 50%, ranging from 30% to 70%; the expected product size of 80 bp to 200 bp perfect ending with G- or C-rich at the 3’ end.

### Experimental validation for amplification efficiency and polymorphism of the developed SSR primers

250 pairs of primers covering all maize chromosomes synthetized by Shanghai Invitrogen Co., Ltd were used for the validation experiment. The materials selected in this experiment were elite inbred lines cataloged to four major heterotic groups in China. The DNA template were B73, Mo17, 91b30, R18, 1212, 583, 3237, R08, Huangzao Si, Zi330, and S37. Genomic DNA was extracted from two weeks old seedlings employing the modification of a CTAB (cetyltrimethylammonium bromide) DNA extraction protocol. PCR was performed in a reaction mixture of 15 μL, containing 50 ng total genomic DNA for template, 1.5 μL 10 × buffer (Mg^2+^), 2.0 μL dNTP (2.5 mM), 100 nM of each SSR-primer, 2 U Taq polymerase, and ddH_2_O. The C1000 thermal cycler (Bio-rad, Inc., Hercules, CA) was used for amplification with the following protocol: an initial denaturation for 3 minutes at 95°C, 35 cycles of denaturation for 30s at 95°C, annealing for 90 s at 55°C, and an extension for 90 s at 72°C; and a final extension for 10 minutes at 72°C. PCR products were electrophoresed on 6.0% polyacrylamide gel. The PIC value for each SSR marker was calculated via the formula previously described.

## Competing interests

The authors declare that they have no competing interests.

## Authors’ contributions

QJT performed bioinformatic analysis, primer design and tested SSR markers. JL designed, coordinated the study and preparing the manuscript. All authors read and approved the final manuscript.

## Supplementary Material

Additional file 1 The distribution of SSRs on chromosome of maize genome. Click here for file

Additional file 2 The length of SSRs with different types. Click here for file

Additional file 3 346 maize materials, including their name, category, sequencing depth and average read length. Click here for file

Additional file 4 82694 SSRs markers, including their locus, motif and PIC. Click here for file
